# Comparison of detection methods for carbonation depth of concrete

**DOI:** 10.1038/s41598-023-47443-8

**Published:** 2023-11-15

**Authors:** Bei Li, Ye Tian, Guoyi Zhang, Yu Liu, Huiping Feng, Nanguo Jin, Xianyu Jin, Hongxiao Wu, Yinzhe Shao, Dongming Yan, Zheng Zhou, Shenshan Wang, Zhiqiang Zhang, Jin Chen, Xiaodong Chen, Yunjun Lu, Xinyi Li, Jiaxi Wang

**Affiliations:** 1 Nanxun Innovation Institute, Zhejiang University of Water Resources and Electric Power, Hangzhou, 310018 China; 2 College of Civil Engineering and Architecture, Zhejiang University, Hangzhou, 310058 China; 3 School of Landscape Architecture, Zhejiang Agriculture and Forestry University, Hangzhou, 310058 China; 4 Engineering Design and Research Institute of Rocket Force, Beijing, 100011 China; 5 China Construction Fifth Engineering Division Corp.,ltd, Hangzhou, 310030 China; 6 Zhejiang Province Institute of Architectural Design and Research, Hangzhou, 310006 China; 7 The National Key Laboratory of Water Disaster Prevention, Hohai University, Nanjing, Jiangsu 210098 China

**Keywords:** Engineering, Civil engineering

## Abstract

This paper presents comprehensive research of the advantages and applicability of various concrete carbonation detection methods. Employing a combination of Phenolphthalein indicator (PI), Thermogravimetric analysis (TGA), X-ray phase analysis (XRD), Fourier transform infrared spectroscopy (FTIR), and Quantitative calcium carbonate analysis (CA), a detailed comparison to determine the carbonation depth in the partial carbonation zone of concrete specimens is conducted. Among the quantitative analysis methods, CA measures CaCO_3_ content based on chemical reactions, while TGA obtains the concentration distribution of Ca(OH)_2_ and CaCO_3_. Among qualitative analysis methods, XRD tested the intensity distribution of Ca(OH)_2_ and CaCO_3_, while FTIR traced the characteristic peaks of C-O functional groups in a specific spectral range to determine the depth of carbonation of concrete. Results indicate that the depth of carbonation values measured by CA, TGA, XRDA, and FTIR are 2–3 times higher than those measured by PI. This research may provide valuable insights for the design of carbonation detection in concrete.

## Introduction

Carbonation is one of the main reasons for the durability deterioration of concrete. Almost all concrete materials will subject to carbonation during their service life due to the presence of CO_2_ from environment. In last decades, carbonation has been widely discussed among the durability investigations of concrete. In the investigation on carbonation of concrete, it is particularly important to accurately measure and evaluate the carbonation depth of concrete. As a result, Phenolphthalein indicator, Thermogravimetric analysis, Fourier transform infrared spectroscopy, Raman spectroscopy, Nuclear magnetic resonance spectroscopy and X-ray phase analysis have been developed and applied for characterizing the carbonation of concrete.

Typically, traditional method in detecting concrete carbonation is a colorimetric method based on phenolphthalein indicator (PI), also known as pH detection^1–5^. But the pH detection has to be operated manually which will introduce manual errors to the detection results. Therefore, Jeong-Il et al.^6^ used the image-processing technique to detect carbonation regions of concrete sprayed with pH indicator. However, pH detection is essentially unable to accurately determine partial carbonation zones^7,8^. Renjie et al.^9,10^ used the width of the carbonation zone to evaluate the carbonation degree of concrete, which was more accurate than pH indicators. To understand the carbonation process comprehensively, it is necessary to delineate the partial carbonation zone and measure the carbonation depth of concrete accurately. As a result, Thermogravimetric analysis (TGA), Fourier transform infrared spectroscopy (FTIR), Raman spectroscopy (RS), infrared spectroscopy (IRS) and X-ray phase analysis (XRD), etc. have been widely applied to detect the carbonation depth of concrete. Thermogravimetric analysis obtains the composition and properties of substances by measuring the change in its weight with increasing temperature. The peaks of the differential curve obtained from TGA test correspond to the carbonation products generated during carbonation process and the integral area state the mass of the carbonation products. Therefore, it is also widely used to quantitatively characterize the carbonation reactions of concrete^11–13^. Be different from thermogravimetric analysis, infrared spectroscopy and X-ray phase analysis determine the material compositions by infrared light or X-ray. The working principle of Fourier transform infrared spectroscopy is to characterize the carbonation of concrete by referencing the characteristic peak of the C–O stretching bonds^14,15^. It was used by Lee et al.^16^ to determine the carbonization depth of concrete. Similarly, Raman spectroscopy was also utilized by Mi et al. to quantify the spectrum intensity variation due to the decrease of Portlandite and the increase of Calcite during the carbonation of concrete^17^. And its experimental results were compared to thermogravimetric analysis, which proved the feasibility of Raman spectroscopy detection. Other than carbonation products recognition, X-ray phase analysis is helpful to state the influence of temperature and relative humidity on carbonation^18,19^. In addition, Géraldine et al.^20^ proposed a new and efficient method to profile drying and carbonation state in concrete by γ-densimetry. Recently, The T2 lifetime and signal intensity behavior in the carbonated and non-carbonated zones of concrete were analyzed by Floriberto et al.^21^ using the NMR spectroscopy.

However, as the theoretical principle of each detection methods is not the same, the measuring results also exist remarkable difference. This research tends to provide a systematic and comprehensive evaluation of common methods for determining the depth of carbonation of concrete. To achieve this object, the principles, test procedures and measurements of the methods for PI, CA, FTIR, XRD and TGA were explored. In addition, the depth carbonation of concrete samples with different water to cement ratios under different time were tested by the five methods. A comparison on the determination procedures and data from the different methods were conducted to assess their applicability and limitations.

## Preparation of carbonized concrete specimens

### Materials and mix proportions

In this research, the P·O 52.5 grade ordinary Portland cement produced by Hubei Huaxin Cement Plant was used. The alkali content (Na_2_O + 0.658K_2_O) of cement was no more than 0.60%, and the fineness was 350 m^2^/kg. The mineral composition of cement is shown in Table 1.

**Table 1:** Mineral composition of cement.

Mineral composition	3CaO∙SiO_2_	2CaO·SiO_2_	3CaO·Al_2_O_3_	4CaO·Al_2_O_3_∙Fe_2_O_3_
Content (%)	55.5	19.1	6.5	10.1

Two kinds of concrete with the water to cement ratio of 0.47 and 0.57 were prepared. The fine aggregate was river sand with fineness modulus of 2.92 and the coarse aggregate was crushed limestone with continuous grading ranging from 5 to 20 mm. The water was tap water. The mix proportions of concrete are shown in Table 2.

**Table 2:** Mix proportions of concrete (kg/m^3^).

Concrete	Water to cement ratio	Cementitious materials	River sand	Crushed stone	Water
HP0.47	0.47	378	709	1133	178
HP0.57	0.57	378	610	1133	215

### Preparation of carbonized concrete specimens

The carbonation test was carried out following the standard of Test Method for the Long-term Performance and Durability of Ordinary Concrete (GB/T 50082-2009)^22^. For each kind of concrete, three specimens with the size of 100 × 100 × 400 mm were prepared. The four side surfaces of each specimen were sealed the wax, and the top and bottom surfaces were left for carbonation. After sealing with wax, the specimens were put into a programmable carbonation chamber for carbonation. During the test, the CO_2_ concentration, relative humidity and temperature were set as 20 ± 3%, 70 ± 5% and 20 ± 2 °C respectively.

At 14 days and 28 days after carbonation, the specimens were taken out of the chamber. A slice with the thickness of 50 mm was cut from each specimen along the length direction. And the cutting surface of each specimen was sealed with paraffin wax. Then, all specimens were placed in the test chamber for further carbonation. The concrete slice was also evenly cut into two thinner parts, with a thickness of 25 mm in each. One slice sample was prepared for Phenolphthalein indicator analysis. The other slice sample was used to prepare concrete powder sample for Quantitative calcium carbonate analysis, Fourier transform infrared spectroscopy analysis, X-ray phase analysis and Thermogravimetric Analysis. In this research, the slice sample was ground into powders along its depth direction by HDM-150 concrete grinding machine. The powder sample was collected when the slice sample was ground off every 1 mm. Then, the powder sample was placed in sealed plastic bottles immediately against reaction with CO_2_ from the air. The collected powder samples were denoted as HPX-Y-Z, in which, X represents water to cement ratio, Y represents the carbonation age and Z represents the test depth.

## Carbonation depth detection methods

### Phenolphthalein indicator

#### Test principle

The carbonation reaction can lead to a continuous drop of pH in concrete from 13 to about 9^23^. As the phenolphthalein is a red organic acid which becomes transparent once pH value is lower than 8.2, so the phenolphthalein can visually distinguish the low-alkaline region from the high-alkaline region. Therefore, the phenolphthalein indicator is always applied to detect the depth of carbonation on the surface of the concrete^24–27^. For the determination of carbonation depth, phenolphthalein indicator is intuitive, convenient and economical.

#### Test procedure

Before the carbonation depth detection, the test section was clean. On one edge of the test section, ten measuring points were marked with a marker in an interval of 10 mm. On the opposite edge of the section, ten measuring points were also marked in the same way. Then, the test section was uniformly sprayed with phenolphthalein indicator with the concentration of 1% (1 g phenolphthalein and 99 g anhydrous ethanol). After 30 s, the perpendicular distance from each measuring point to the color separation line was measured with a ruler. The depth of carbonation of the concrete specimen was taken as the average of the perpendicular distance.

### Quantitative calcium carbonate analysis

#### Test principle

When the carbonation product, CaCO_3_, reacts with hydrochloric acid, CO_2_ gas will be released. Within a sealed container, the released CO_2_ may induce the increase of air pressure. The corresponding air pressure growth can be used to calculate the CaCO_3_ content in the sample^28^. In this study a precision concrete carbonation meter, type DRB-C1, was used to determine the amount of CaCO_3_ in the concrete. It can quantitatively reflect the carbonation degree of concrete.

#### Test procedure

**Figure 1:**
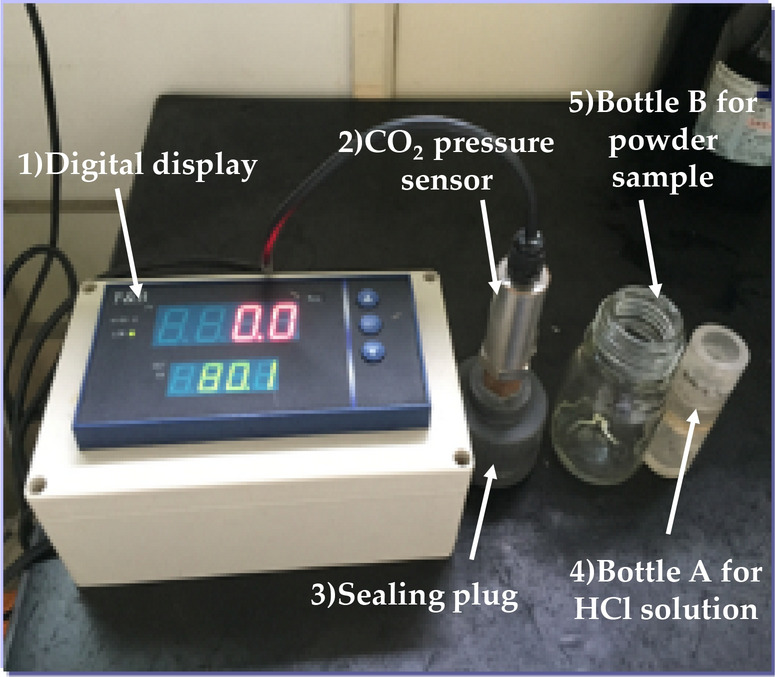
Test device.

A carbonation meter was used to measure the CaCO_3_ content in the powder sample. As shown in Fig. 1, the carbonation meter consists of five main components, including digital displayer, CO_2_ pressure sensor, sealing plug, Bottle A for HCl solution and Bottle B for powder sample.

The influence of temperature on gas pressure was taken into account and the entire test procedure was carried out under 20 ± 2 °C. Before detection, the sensor cable and the digital displayer were interconnected and the digital readout was checked for proper auto-zeroing. Then, the sensor threads were tightly connected to the sealing plug. The concrete powder with the weight of 1 g was placed in bottle B. About 20 ml of HCl solution with the concentration of 1 mol/l was put into bottle A using a dropper.

Bottle A with HCl solution was laid down horizontally and installed on the sealing plug. Bottle B with powder sample was also screwed on the sealing plug horizontally over bottle A. Then, the sealing plug, bottle A and B were placed vertically on HY-4 T speed regulated multi-purpose oscillator for oscillating under 120 Hz for 5 min. During oscillating, the HCl solution in bottle A chemically reacted with the concrete powder sample in bottle B and the air pressure increased gradually. After a full reaction between them, the air pressure value on the digital display kept unchanged. Then the air pressure was recorded. The mass fraction of CaCO_3_ in concrete powder was indexed from a table according to the pressure value. Each concrete powder sample was measured three times and average value was taken as the measured result at the corresponding depth. Then the mass fraction of CaCO_3_ in concrete was plotted against the depth of the concrete specimen. By this way, the quantitative calcium carbonate analysis method may provide a quantitative description of the concrete carbonation.

### Fourier transform infrared spectroscopy

#### Test principle

Fourier transform infrared spectroscopy is a method that enables the species of a substance to be inferred from the molecular vibrations of specific groups^29–32^. During the carbonation of concrete, the C=O bond in CO_2_ changes to C–O bond in CaCO_3_. Therefore, the depth of carbonation can be measured from the footprint of the C–O characteristic peaks corresponding to the baseline with a spectrum ranging from 1410 to 1510/cm. The peak bands are characterized by infrared absorbance.

#### Test procedure

In this study, pyrolysis gas chromatography coupled with Fourier transform infrared spectroscopy was utilized to detect the C–O bonds in concrete. The test device was Nicolet 5700, which was manufactured by SGE Australia Pty. Ltd. The infrared spectrum ranged from 4000 to 400/cm.

The Fourier transform infrared spectroscopy detection included following steps. At first, about 0.5 mg of the solid sample was taken and the solid sample was well ground under an infrared lamp. Afterwards, approximately 50 mg of dried KBr powder was mixed and the grinding started again until the mixture was homogeneous. To avoid the influence of scattered light, both the sample and the KBr powder should be dried and ground until the diameter of each particle was less than 2 µm. The mixed powder was evenly put in a mould into a thin layer. The test sample was then pressed under 0.8–1 GPa by hydraulic press for 2–5 min. Then, the powder sample was pressed to a transparent sheet for detection. The background spectrum of the laboratory environment was scanned before the test. The prepared sample was placed to the chamber of the instrument and the infrared spectrum of the sample was recorded.

### X-ray phase analysis

#### Test principle

**Figure 2:**
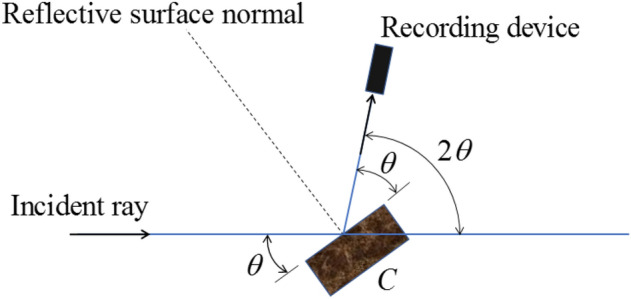
Schematic diagram of X-Ray diffraction principle.

The working principle of X-ray diffraction is shown in Fig. 2. If the angle of incidence satisfies Bragg's law, as demonstrated in Eq. (1), the X-ray intensity is enhanced by diffraction. As a result, the crystalline composition of the material can be determined from the diffraction angles. At the same time, the intensity of the diffracted rays depends on the number of crystals in the material. Therefore, the relative proportion of the diffracted ray peaks can be used to qualitatively determine of the chemical composition of the material.

1 $$ 2d\sin \theta = n\lambda $$

where *d* is the lattice spacing; *θ* is the incidence angle; *n* is an arbitrary integer, stated as the diffraction level; *λ* is the wavelength.

**Figure 3:**
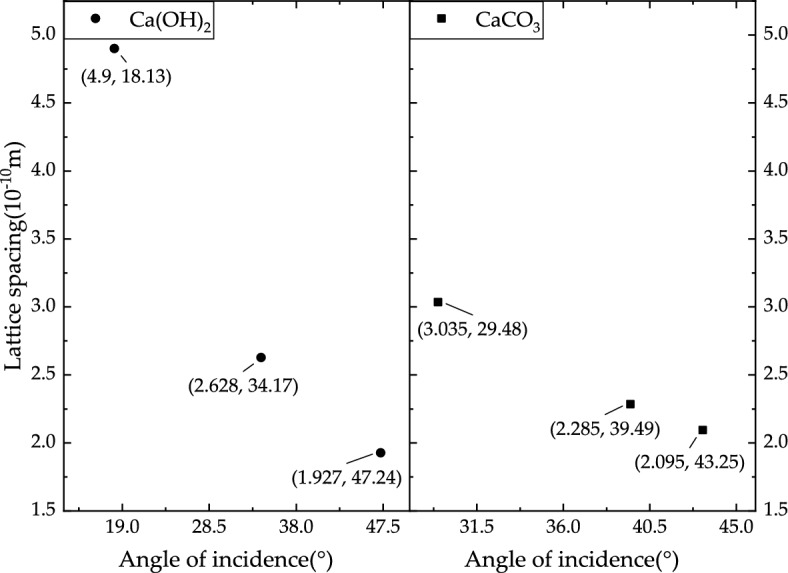
Lattice spacing and incident angle of CaCO_3_ and Ca(OH)_2_.

The crystal lattice spacing of Ca(OH)_2_ is 3.035, 2.095 and 2.285 Å, while the lattice spacing of calcium carbonate is 2.628, 4.9 and 1.927 Å. As shown in Fig. 3, the incident angles of calcium hydroxide and calcium carbonate are 29.48° and 18.13°, respectively. Furtherly, the diffraction peaks can be identified from the X diffraction detection results.

#### Test procedure

The XRD analysis was carried out on a Shimazu Model XD-3 X-ray diffractometer. During the test, the concrete powder was dried in a dryer at 105 °C for 2 min. A 5 mg powder sample was furtherly ground until the particle size was about 40 μm. The powder sample was put into a groove of the sample table until the groove was fully filled. The excess powder was scraped off so that the surface of filled powder sample was flush with the surface of sample table. Then an X-ray diffraction analysis instrument was used to obtain the spectra of the powder X-ray diffraction analysis and the relative diffraction peaks of CaCO_3_ and Ca(OH)_2_ were obtained. The operating voltage and current of CuKa laser radiation was 40 kV and 100 mA. The scan rate was 4°/min. The scan ranged from 10 to 80°.

### Thermogravimetric analysis

#### Test principle

**Figure 4:**
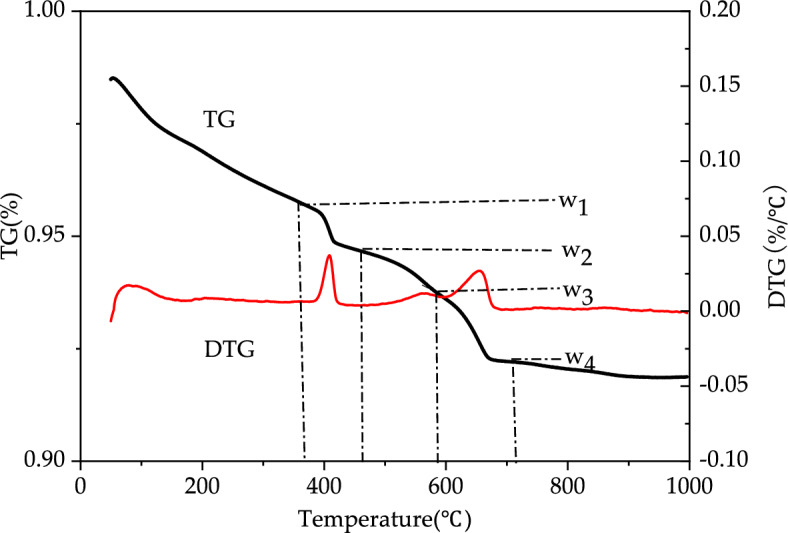
The concrete characteristic curves of thermogravimetric analysis with temperature^36^.

Thermogravimetric analysis is a method for measuring the mass of a substance through the decomposition temperature^33,34^. In concrete, CaCO_3_ may decompose into CaO and CO_2_ within the temperature band from 580 to 710 °C. Therefore, CaCO_3_ content of the sample can be deduced from the mass loss caused by CO_2_ release^35,36^, which would serve the purpose of quantifying the degree of carbonation of concrete. When the temperature of the concrete specimen in the thermogravimetric analysis (TGA) apparatus increases from 0 to 1000 °C, the mass loss of the specimen is shown in Fig. 4^37^. There were some differences in the definition of the decomposition temperature intervals for Ca(OH)_2_ and CaCO_3_ between Liu^38^ and Short et al.^39^. During thermogravimetric analysis, the sample undergoes mass changes under programmed temperature control allowing quantitative analysis of the components of the specimen.

#### Test procedure

A thermogravimetric analyser-Q500 was used in this study. During the experiment, the powder sample was baked in an oven. Then a sieve with mesh of 0.3 mm was used to give good thermal conductivity during the experiment. After sieving, the sample was put into a crucible and placed in the thermogravimetric analyzer. The decomposition temperature was set to increase from 20 to 1000 °C at a rate of 10 °C per minute. The final mass loss of the specimen was obtained and recorded as a function of temperature.

## Results and discussion

### Results of phenolphthalein indicator

**Figure 5:**
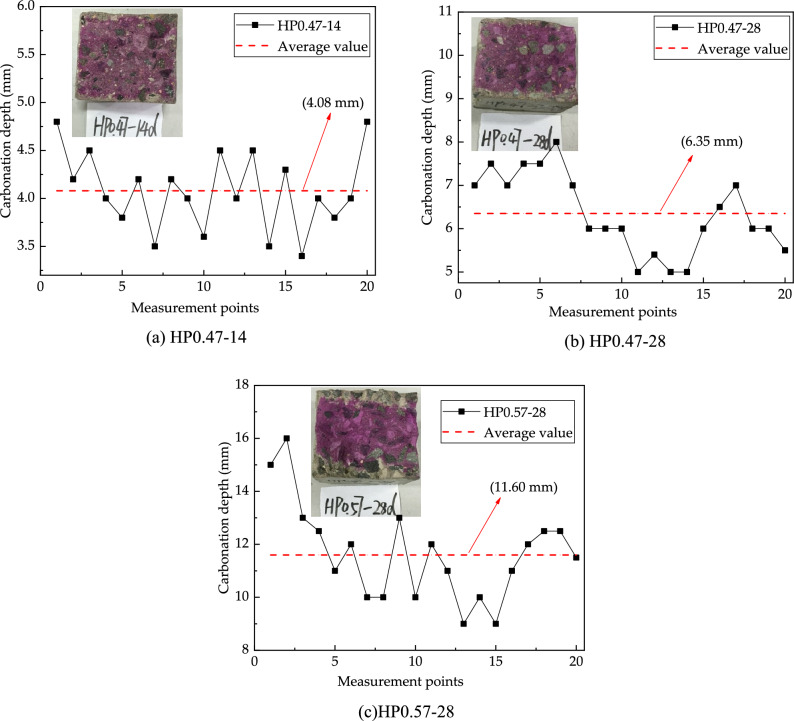
Depth of carbonation of the specimen tested with phenolphthalein indicator.

Figure 5 shows the carbonation depth of concrete specimen detected with phenolphthalein indicator. For concrete HP0.47, the average carbonation depths at 14 days and 28 days are 4.08 and 6.35 mm, respectively. For concrete HP0.57, the average carbonation depth on 28 days is 11.60 mm. It is reasonable to find out that the carbonation depth is higher under a longer carbonation age and a higher water to cement ratio. However, the carbonation depths fluctuate and are non-uniformly distributed along the depth direction of the specimens. The carbonation depth values determined by Phenolphthalein indicator are not uniform since the distribution of coarse aggregates in concrete affects the carbonation process of concrete.

### Results of calcium carbonate quantitative analysis method

**Figure 6:**
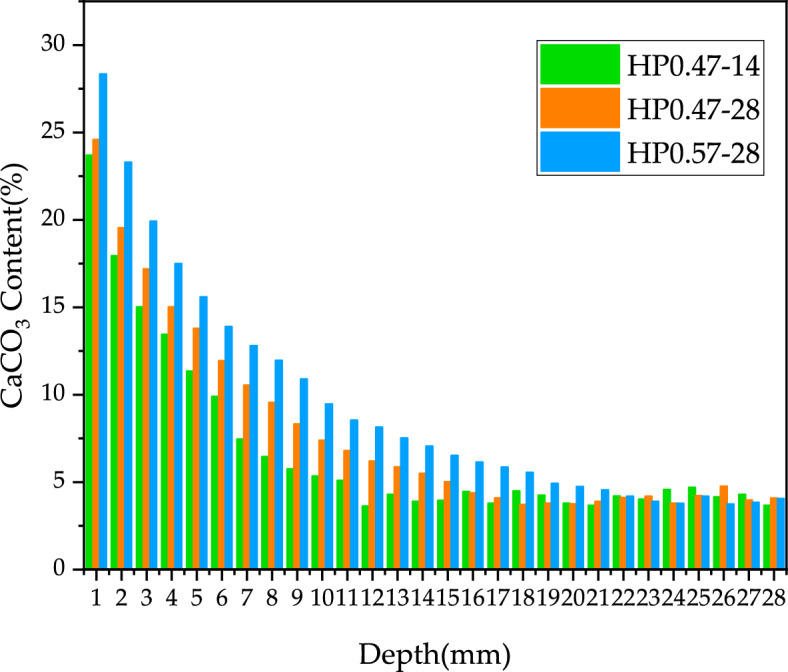
Test results of CaCO_3_ content under different depths (%).

The distribution of CaCO_3_ content along the depth of concrete specimen for HP0.47-14, HP0.47-28 and HP0.57-28 is shown in Fig. 6. With the increase of sample depth, the CaCO_3_ content decreases at first and then tends to be stable. It is obvious that the decrease in CaCO_3_ content is attributed to the lower carbonation degree of concrete as the depth of the sample increases. While Fig. 6 indicates that the CaCO_3_ content can be recognized deep in concrete specimen. Actually, this phenomenon does not mean that carbonation occurs deep in concrete during the experiment. As the coarse aggregate of the concrete was limestone which was mainly composed of CaCO_3_, the powder sample contained a small amount CaCO_3 ground_ from coarse aggregate. In carbonation studying, the complete carbonation zone is defined as the alkaline substances in concrete, such as Ca(OH)_2_, is completely carbonized into CaCO_3_. Based on this definition, the CaCO_3_ distribution in Fig. 6 indicates that there is no complete carbonation zone in all three kinds of concrete. The absence of a complete carbonation zone can be attributed to the short carbonation age and none of the three types of concrete is fully carbonized.

**Figure 7:**
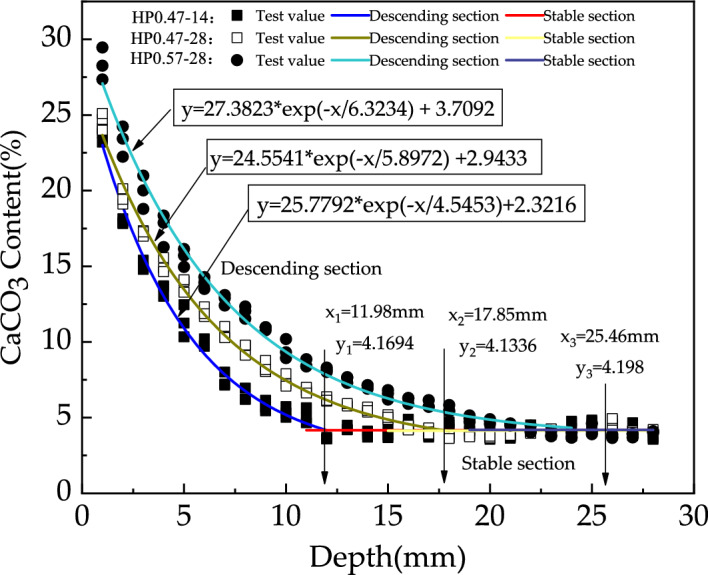
Fitting curves of CaCO_3_ content of HP0.47-14, HP0.47-28 and HP0.57-28.

In this research, the CaCO_3_ content in descending section is numerically fitted using an exponential function and the CaCO_3_ content in the stable section is assumed to be a constant. The fitted curves for different concretes are shown in Fig. 7. Theoretically, the intersection point of the two fitting curves indicates the front of carbonation reaction zone. So, the carbonation depth of concrete can be mathematically assigned as the depth of intersection point. As demonstrated in Fig. 7, the carbonation depths of HP0.47-14, 0.47-28 and 0.57-28 are 11.98 mm, 17.85 mm and 25.46 mm respectively.

It can be seen from Fig. 7 that the carbonation depths detected by calcium carbonate quantitative analysis and phenolphthalein indicator show similar trend under the influence of water to cement ratio and carbonation age. While the quantitative calcium carbonate analysis method provides a detailed determination of calcium carbonate content.

### Results of Fourier transform infrared spectroscopy

**Figure 8:**
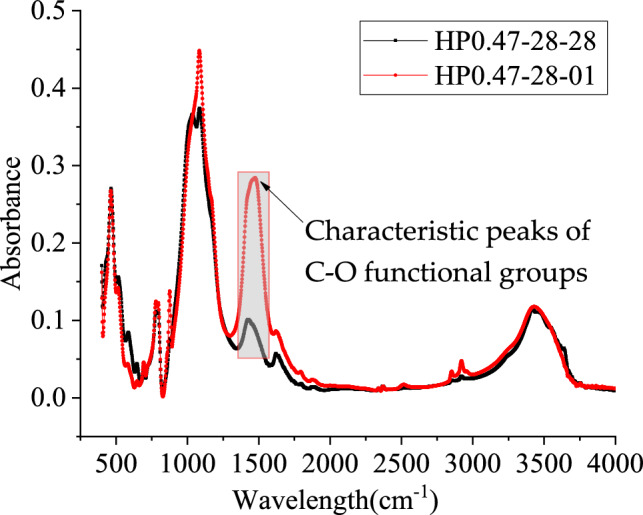
The results of FTIR mapping of test specimenHP0.47-28 at 1 and 28 mm.

**Figure 9:**
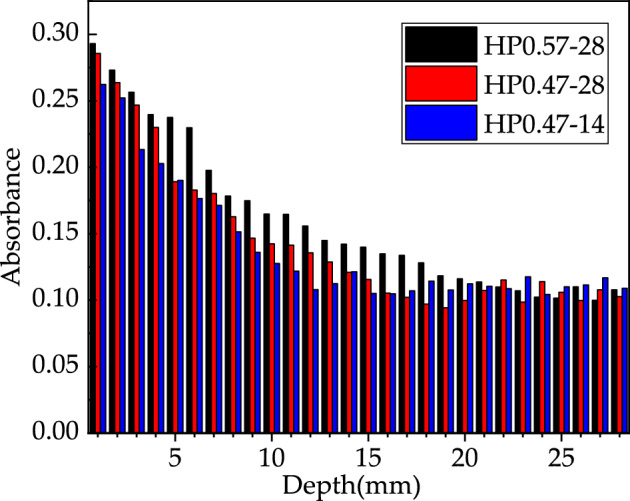
The change of characteristic peak of the C–O functional group with depth detected by FTIR.

Taking the specimen HP0.47-28 as an example, Fig. 8 shows the FTIR spectral results of this specimen at the depth of 1 mm and 28 mm. The results show that the change in the degree of carbonation is closely related to the change in the characteristic peak of the C–O functional group. Figure 9 furtherly shows the changes in the characteristic peaks of C–O functional groups obtained using FTIR detection for HP0.47-14, HP0.47-28 and HP0.57-28 in the depth from 1 to 28 mm. With the increase of depth, the characteristic peak value of the C–O functional group gradually decreases, corresponding to the decrease of CaCO_3_ content. Due to the presence of carbonates in the concrete aggregates, the C–O absorbance values become constants at a certain depth.

**Figure 10:**
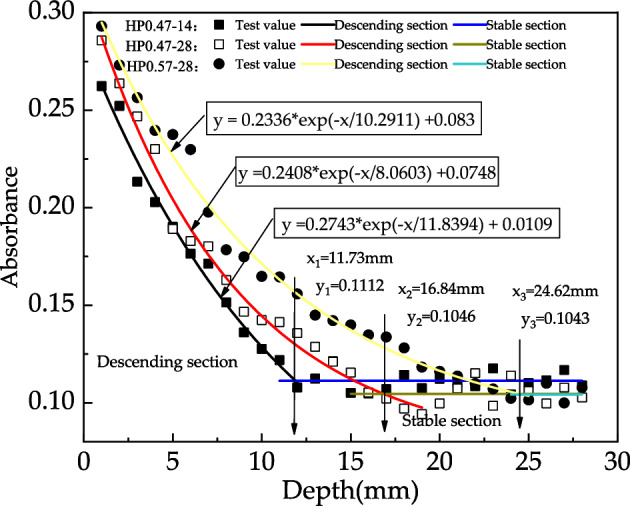
The fitted curves of characteristic peak of the C–O functional group of HP0.47-14, HP0.47-28 and HP0.57-28.

Figures 10 shows the fitted curves of characteristic peak of the C–O functional group of HP0.47-14, HP0.47-28 and HP0.57-28. The exponential and linear functions are also applied to characterize the descending and stable sections respectively. The intersection of two curves can be identified as the carbonation boundary and the depth of the carbonation reaction zone. According to Fig. 10, the carbonation depths HP0.47-14, HP0.47-28 and HP0.57-28 are 11.73 mm, 16.84 mm and 24.62 mm, respectively.

### Results of X-ray phase analysis

**Figure 11:**
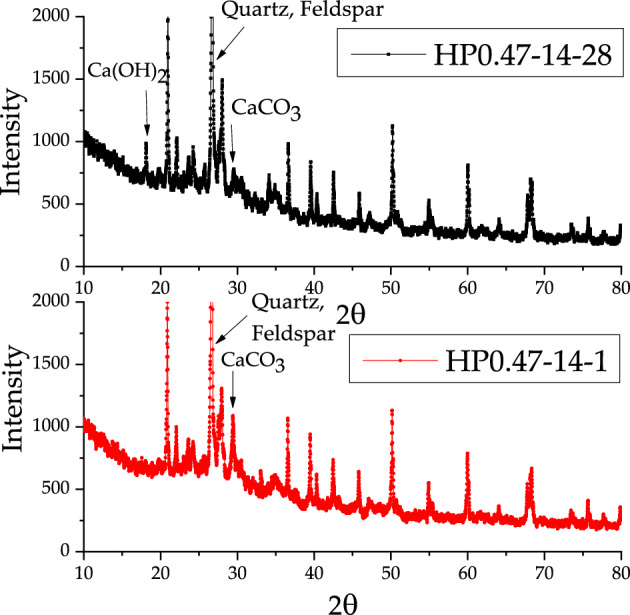
X-Ray diffraction analysis of HP0.47-14-1 and HP0.47-14-28.

**Figure 12:**
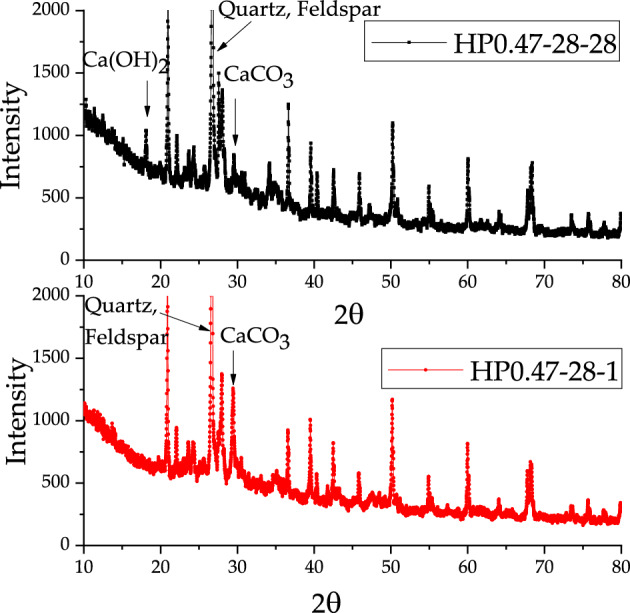
X-Ray diffraction analysis of HP0.47-28-1 and HP0.47-28-28.

**Figure 13:**
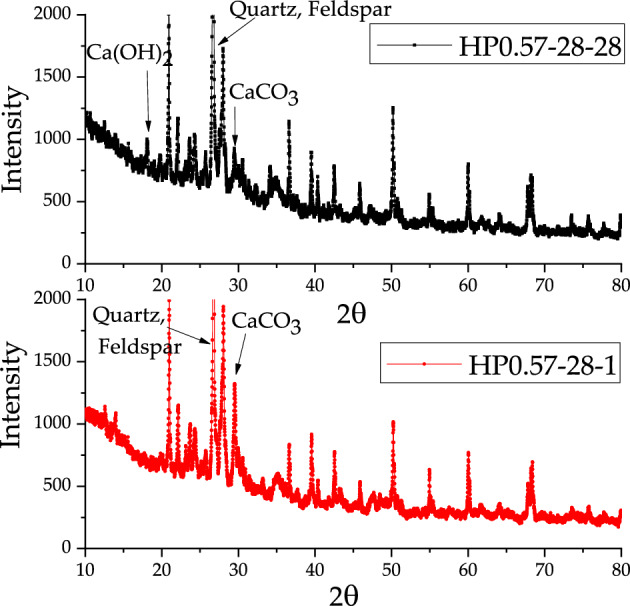
X-Ray diffraction analysis of HP0.57-28-1 and HP0.57-28-28.

The X-ray test results show the four main crystal substance in the concrete, such as quartz, feldspar, Ca(OH)_2_ and CaCO_3_. In these crystals, quartz and feldspar came from the aggregate. However, the content of Ca(OH)_2_ and CaCO_3_ varied slightly during the carbonation of concrete specimens. Figures 11, 12 and 13 present the differences between the X diffraction patterns at the depths of 1 mm and 28 mm for three kinds of concrete, respectively. The diffraction peak of calcium carbonate at the depth of 1 mm was large, while no diffraction peak of calcium hydroxide can be found. In contrast, at the depth of 28 mm, diffraction peaks of both calcium hydroxide and calcium carbonate could be recognized.

**Figure 14:**
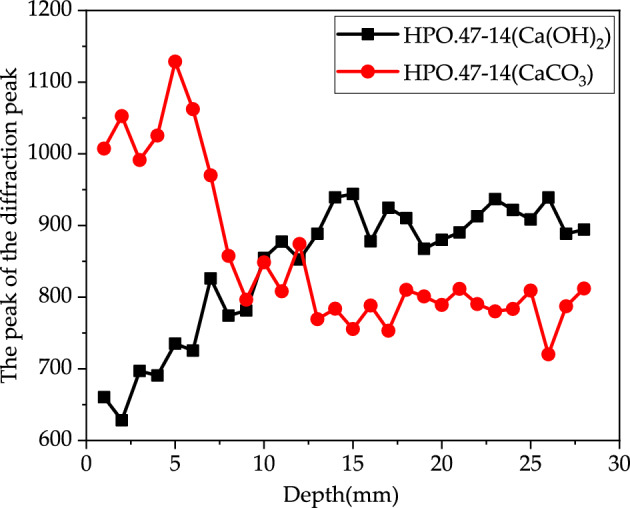
The development of X-Ray diffraction peak on Ca(OH)_2_ and CaCO_3_ of HP0.47-14.

**Figure 15:**
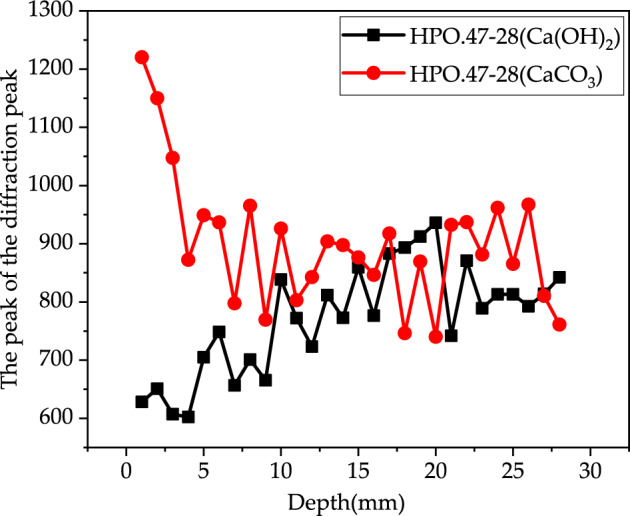
The development of X-Ray diffraction peak on Ca(OH)_2_ and CaCO_3_ of HP0.47-28.

**Figure 16:**
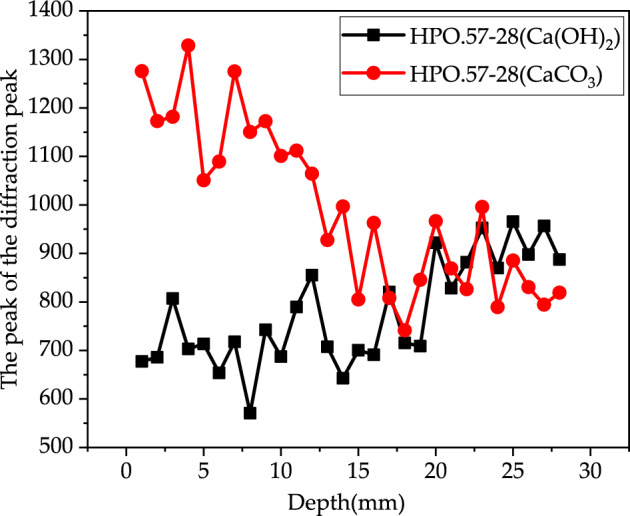
The development of X-Ray diffraction peak on Ca(OH)_2_ and CaCO_3_ of HP0.57-28.

Figures 14, 15 and 16 show the development of X-Ray diffraction peak of Ca(OH)_2_ and CaCO_3_ with depth for HP0.47-14, HP0.47-28 and HP0.57-28. On the surface of the sample, the X-Ray diffraction peak value of CaCO_3_ is much higher than Ca(OH)_2_. From the depth of 1–28 mm, the CaCO_3_ diffraction peak value of CaCO_3_ tends to decrease until it becomes relatively stable. While the peak value of Ca(OH)_2_ tends to increase before it reaches to platform. The distribution of CaCO_3_ can also be divided into descending section and stable section too. But it can be seen from Figs. 14, 15 and 16 that the peak values fluctuate dramatically within the depth of concrete specimen, which make it very difficult to obtain the fitting curves of the two sections mathematically.

Generally, the complementary correspondence of the diffraction peaks of CaCO_3_ and Ca(OH)_2_ can be deduced from Figs. 14, 15 and 16. But it cannot precisely recognize the transition point from the descending to the stable section. As a result, XRD can only be used as an auxiliary qualitative tool in the characterization of carbonation properties.

### Results of thermogravimetric analysis

**Figure 17:**
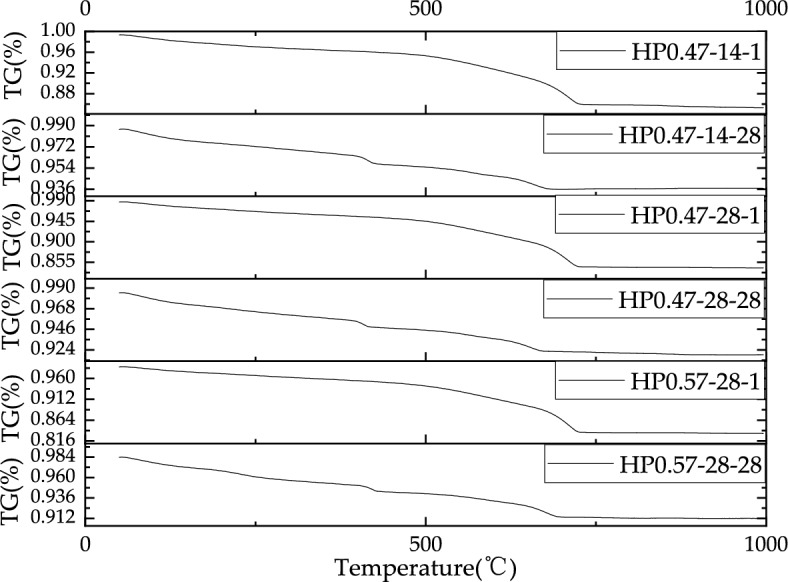
The TG profiles of inner and outer layers of HP0.47-14, HP0.47-28 and HP0.57-28.

**Figure 18:**
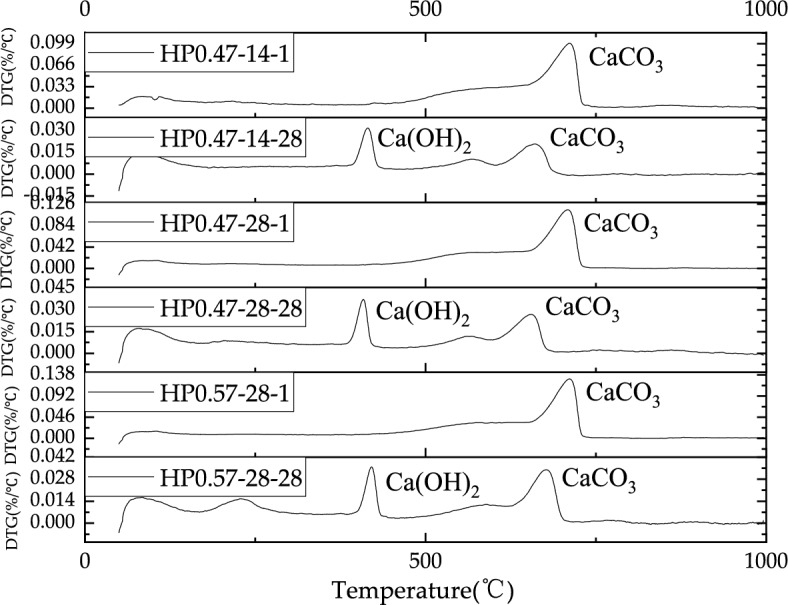
The DTG profiles of inner and outer layers of HP0.47-14, HP0.47-28 and HP0.57-28.

Figures 17, 18 show the TG and DTG profiles of HP0.47-14, HP0.47-28 and HP0.57-28 at the depth of 1 mm and 28 mm respectively. At the depth of 1 mm, all the three samples show significant mass loss of CaCO_3_ at temperatures between 700 and 750 °C, while no Ca(OH)_2_ decomposition can be found. In contrast, the samples taken from the depth of 28 mm for all three kinds of concrete clearly show two decomposition peaks representing CaCO_3_ and Ca(OH)_2_. This also proves that the specimen at 1 mm is highly carbonated with a high CaCO_3_ content.

**Figures 19:**
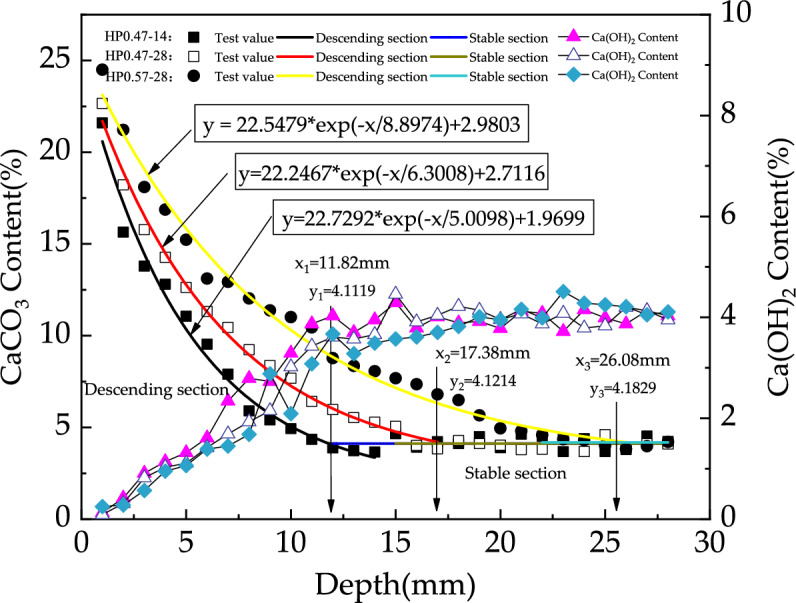
Distribution of CaCO3–Ca(OH)_2_ content in HP0.47-14, HP0.47-28 and HP0.57-28 and the fitted curves of the characteristic peaks of C–O functional groups.

Figure 19 shows the variation in CaCO_3_ content with increasing depth for HP0.47-14, HP0.47-28 and HP0.57-28. The decreasing and stable section of CaCO_3_ content are characterized by fitting exponential and linear functions, respectively. The intersection point between the two sections is used as the carbonation depth. For HP0.47-14, HP0.47-28 and HP0.57-28, the carbonation depths are 11.82 mm, 17.38 mm, 26.08 mm respectively.

As can be seen from Fig. 19, the fully carbonation zone of the test block was not reflected. There was also 3.5% to 5% CaCO_3_ present in the stable zone. The main reason for this may be the presence of CaCO3 in the original specimen powder, since the composition of concrete aggregate contains calcium carbonate, calcium silicate and silicon dioxide.

As shown in Fig. 19, there exists 4% Ca(OH)_2_ at the depth of 28 mm. Even if this part of Ca(OH)_2_ is completely carbonated, the generated CaCO_3_ is about 5.4% which is still lower than the CaCO_3_ content measured during the experiment. This proves that there were more substances involved in the carbonation reaction than Ca(OH)_2_.

## Comparison of test results and applicability analysis of test methods

### Comparison of test results

**Figure 20:**
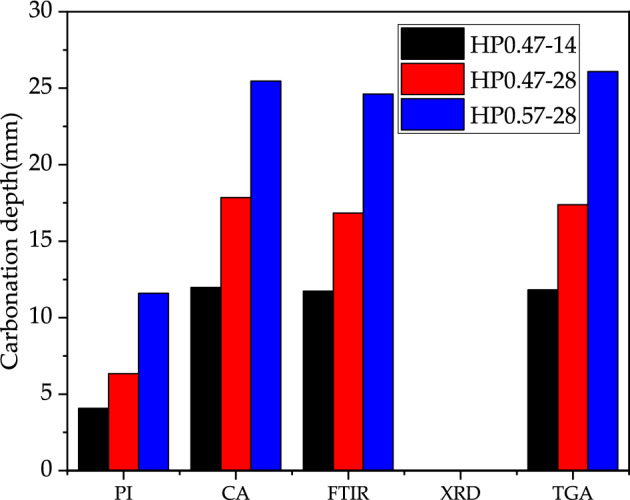
The carbonation depth values determined by the five methods.

Figure 20 shows the carbonation depths determined by the five methods. Among them, X-ray physical analysis (XRD) was unable to give a quantitative depth of carbonation. The results of the quantitative calcium carbonate analysis (CA), Fourier transform infrared spectroscopy (FTIR) and thermogravimetric analysis (TGA) were closer, but all were much greater than the phenolphthalein indicator (PI).

Table 3 shows the carbonation depths ratio of quantitative calcium carbonate analysis (CA), infrared spectroscopy (FTIR) and thermogravimetric analysis (TGA) to phenolphthalein indicator (PI). Table 3 indicates that the carbonation depth value determined by CA, IR or TGA is 2–3 times greater than that obtained by PI.

**Table 3:** The proportional relationship of carbonation depth by different methods.

	CA/PI	FTIR/PI	TGA/PI
HP0.47-14	2.936	2.875	2.897
HP0.47-28	2.811	2.652	2.737
HP0.57-28	2.195	2.122	2.248

### Applicability analysis of test methods

**Figure 21:**
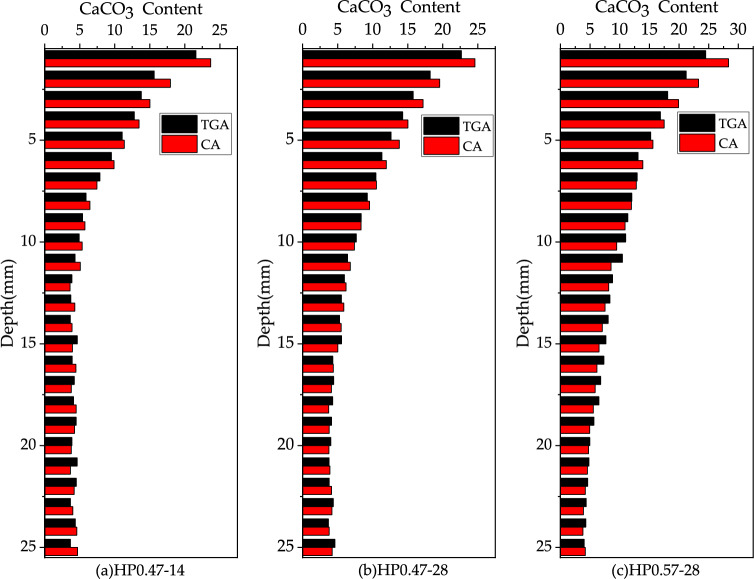
Comparison of the CaCO_3_ content determined by CA and TGA.

**Figure 22:**
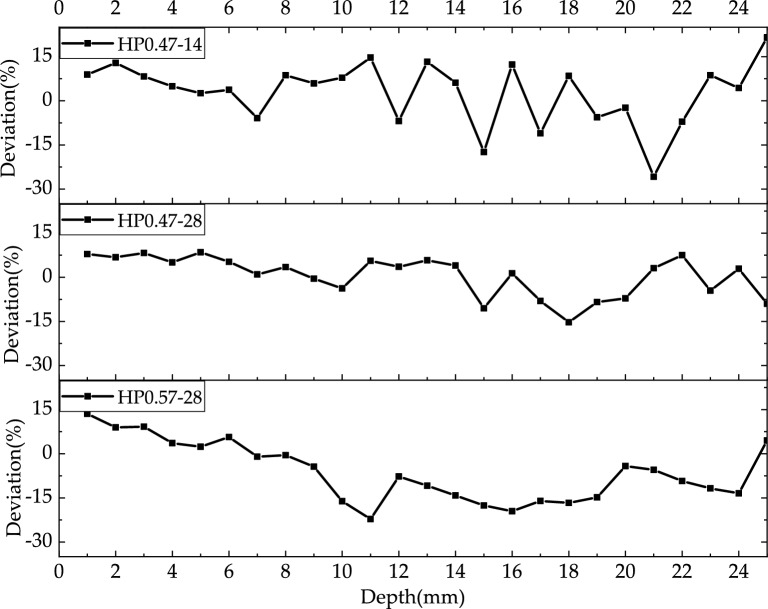
Deviation data for different depths of CA and TGA.

The above five methods in determining the degree of carbonation can be divided into two categories. The first type of quantitative determination methods includes CA and TGA. The second type of qualitative analysis methods are IR, XRD and PI. Figure 21 shows the comparison of the CaCO_3_ content determined CA and TGA. Figure 22 shows deviation data for different depths of CA and TGA. The data deviation was largely within 15% and the results were more approximate.

However, TGA suffers from a lack of measurement accuracy due to the unclear temperature limits for the decomposition of each substance during high temperature heating. The calcium carbonate content of specimen HP0.47-28, for example, is greater at a depth of 15 mm than at a depth of 14 mm, which is not agree with the theoretical prediction. TGA is relatively complex to prepare samples, which is expensive to test and not economical. In contrast, the economy, accuracy and applicability of CA is strongly recommended.

In carbonation detection, the generation of CaCO_3_ or the consumption of Ca(OH)_2_ is applied to evaluate the carbonation degree of concrete.

Therefore, PI can not essentially reflect the carbonation of the concrete. The uncertainty of PI is compensated to some extent by XRD and IR. However, XRD is not precise enough and can only distinguish between specimens with a remarkable difference in calcium hydroxide or calcium carbonate content. IR allows precise calculation of the depth of the carbonation reaction zone. Nonetheless, infrared spectroscopy is characterized by absorbance, and it is not possible to obtain the content of carbonation products at different depths.

## Conclusions

In this paper, five methods, namely PI, CA, IR, XRD and TGA, were used for the experimental study. They were used to determine the carbonation properties of concrete specimens with different water-cement ratios and different carbonation ages. All five test methods can be used to determine the depth of concrete carbonation, each with its own advantages and disadvantages. The following conclusions can be drawn:

1. PI is easy to operate and easy to use in engineering practices. However, the results cannot accurately determine the degree of concrete carbonation.
2. CA is highly accurate and effectively determines the amount of carbonation products at each depth. It accurately delineates the depths of fully carbonation reaction zone, uncarbonation reaction zone and partial carbonation reaction zone.
3. IR calculates the depth of carbonation, but cannot get the content of carbonation products at a certain point. XRD cannot do for the quantitative measurement of calcium carbonate or calcium hydroxide. TGA lacks precision due to the uncertainty of the temperature limits for the decomposition of each substance during high temperature heating. IR, XRD and TGA are more complicated to produce samples, costly in terms of manpower and financial resources and not economical enough.
4. The test results obtained by the CA, IR and TGA are relatively similar. The measured carbonation depth values were 2–3 times higher than those obtained by the PI. The test results of the quantitative calcium carbonate analysis method are more accurate and reliable. It effectively determines the depth of concrete carbonation, which is worth promoting.
5. As the age of carbonation continues, the depth of carbonation increases, and the greater the water to cement ratio at the same age, the greater the depth of carbonation.

## Data Availability

The datasets used and/or analysed during the current study available from the corresponding author on reasonable request.
