# Author Correction: Comparison of detection methods for carbonation depth of concrete

**DOI:** 10.1038/s41598-024-55981-y

**Published:** 2024-03-05

**Authors:** Bei Li, Ye Tian, Guoyi Zhang, Yu Liu, Huiping Feng, Nanguo Jin, Xianyu Jin, Hongxiao Wu, Yinzhe Shao, Dongming Yan, Zheng Zhou, Shenshan Wang, Zhiqiang Zhang, Jin Chen, Xiaodong Chen, Yunjun Lu, Xinyi Li, Jiaxi Wang

**Affiliations:** 1 Nanxun Innovation Institute, Zhejiang University of Water Resources and Electric Power, Hangzhou, 310018 China; 2 College of Civil Engineering and Architecture, Zhejiang University, Hangzhou, 310058 China; 3 School of Landscape Architecture, Zhejiang Agriculture and Forestry University, Hangzhou, 310058 China; 4 Engineering Design and Research Institute of Rocket Force, Beijing, 100011 China; 5 China Construction Fifth Engineering Division Corp.,ltd, Hangzhou, 310030 China; 6 Zhejiang Province Institute of Architectural Design and Research, Hangzhou, 310006 China; 7 The National Key Laboratory of Water Disaster Prevention, Hohai University, Nanjing, 210098 Jiangsu China

Correction to: *Scientific Reports* 10.1038/s41598-023-47443-8, published online 15 November 2023

The original version of this Article contained errors in the Affiliations.

Bei Li was incorrectly affiliated with ‘College of Civil Engineering and Architecture, Zhejiang University of Water Resource and Electric Power, Hangzhou 310018, China’.

The correct affiliations for Bei Li are listed below.

Nanxun Innovation Institute, Zhejiang University of Water Resources and Electric Power, Hangzhou 310018, China.

College of Civil Engineering and Architecture, Zhejiang University, Hangzhou 310058, China.

Additionally, Xinyi Li was incorrectly affiliated with ‘Geotechnical Institute, Jiangsu Jianke Geotechnical Engineering Investigation and Design Co., LTD, Nanjing 210003, China’.

The correct affiliation for Xinyi Li is listed below.

The National Key Laboratory of Water Disaster Prevention, Hohai University, Nanjing, Jiangsu 210098, China.

In addition, the Acknowledgements section contained errors.

“This work was supported by the National Natural Science Foundation of China (Grant number 52108257, 52278223); Zhejiang Provincial Natural Science Foundation of China (Grant number LGG22E080003); Integrated application of intelligent operation and maintenance technology for Hong Kong-Zhuhai-Macao Bridge (Grant number 2019YFB1600700); Key military projects of rocket force (Grant number GXTC-A1-22780035); Scientific research project of Zhejiang University of Water Resource and Electric Power(Grant number X20210014).”

now reads:

“This work was supported by the National Natural Science Foundation of China (Grant number 52108257, 52278223); Zhejiang Provincial Natural Science Foundation of China (Grant number LGG22E080003, LTGS24E080006); Integrated application of intelligent operation and maintenance technology for Hong Kong-Zhuhai-Macao Bridge (Grant number 2019YFB1600700); Key military projects of rocket force (Grant number GXTC-A1-22780035); Scientific research project of Zhejiang University of Water Resource and Electric Power(Grant number X20210014). The authors would also like to appreciate the strong support of the “Nanxun Young Scholars” project.”

The original Article has been corrected.

